# Improved platelet recovery in cryopreserved platelets reconstituted in freeze‐dried plasma

**DOI:** 10.1111/trf.70129

**Published:** 2026-02-15

**Authors:** Kristina Ehn, Per Sandgren, Klara Asplund Högelin, Agneta Wikman

**Affiliations:** ^1^ Clinical Immunology and Transfusion Medicine Karolinska University Hospital Stockholm Sweden; ^2^ Department of Medicine Huddinge Karolinska Insitutet Stockholm Sweden; ^3^ Department of Laboratory Medicine Karolinska Institutet Stockholm Sweden

## Abstract

**Background:**

Cryopreserved platelets can support availability in settings where fresh platelets are inaccessible. Freeze‐dried plasma (FDP) may serve as an alternative reconstitution medium to fresh frozen plasma (FFP) when plasma thawing is impractical. The effects of FDP on cryopreserved platelet quality remain underexplored. Thus, we aimed to evaluate its suitability.

**Study Design and Methods:**

Cryopreserved platelets were prepared from double‐dose buffy‐coat concentrates, split, prewashed, and frozen using dimethyl sulfoxide (DMSO) (5–6%). After thawing, paired units were resuspended in FFP (AB = 8) or FDP (OctaplasLG Powder, AB = 8). Platelet count and mean platelet volume were analyzed alongside blood gas parameters. Additionally, extracellular lactate dehydrogenase (LDH), sP‐selectin, and VEGF were measured in the supernatant. Platelet markers (aggregation, adhesion, and activation) and microparticles were analyzed by flow cytometry. Clotting ability was evaluated using ROTEM.

**Results:**

After thawing, FDP units contained significantly more platelets than FFP units 206 ± 27 versus 176 ± 18 ×10^9^/units (*p* = .0006). LDH activity was lower (*p* = .040), whereas VEGF levels were higher (*p* = .0003) in the FDP group. Oxygen and carbon dioxide pressure differed significantly, yet pH was normal. Phenotypic expression and microparticle content demonstrated no significant differences. FDP units showed a shorter clotting time in ROTEM EXTEM (50 ± 5 vs. 58 ± 9 s, *p* = .035), although clot strength was similar.

**Discussion:**

FDP‐reconstituted platelets were functionally comparable to those reconstituted with FFP, while demonstrating improved recovery and enhanced clot initiation, potentially due to differences in plasma composition. Given its logistical advantages, particularly in resource‐limited settings, FDP represents a promising reconstitution medium for cryopreserved platelets.

**Abbreviations**
CFT: clot formation time
CT: clotting time
DMSO: dimethyl sulfoxide
FDP: freeze‐dried plasma
FFP: fresh frozen plasma
FLyP: French lyophilized plasma
LDH: lactate dehydrogenase
MCF: maximum clot firmness
MFI: median fluorescence intensity
MPV: mean platelet volume
PMPs: platelet microparticles
ROTEM: rotational thromboelastometry
sP‐selectin: soluble P‐selectin
VEGF: vascular endothelial growth factor
ΔΨm: mitochondrial membrane potential

## 1 INTRODUCTION

Cryopreserved platelets are an alternative to conventionally stored platelets that permit long‐term storage at −80°C.^1^, ^2^ The extended shelf life of up to several years enables blood banks to stockpile platelets, thereby mitigating the challenges of unpredictable platelet supply. The limited storage time of conventional platelets (5–7 days) is particularly problematic in remote or austere environments such as military operations, where cryopreserved platelets provide a valuable alternative.^3^, ^4^, ^5^ Cryopreserved platelets exhibit both phenotypic and functional differences from conventional platelets, including procoagulant properties that render them suitable for treating actively bleeding patients.^5^, ^6^, ^7^

The most widely used cryopreservation method, developed by Valeri, includes 5–6% dimethyl sulfoxide (DMSO) as a cryoprotectant. In this method, platelets are prewashed, and only a small volume (10–30 mL) is frozen.^8^ Upon request, cryopreserved platelets are thawed and resuspended, typically in thawed fresh frozen plasma (FFP), although other liquids such as saline and additive solutions have also been tested.^8^, ^9^, ^10^ Plasma is generally preferred due to its glucose content, which may support platelet recovery, and its supply of coagulation factors essential for managing bleeding.^11^, ^12^ However, thawing FFP takes at least 20 min, which can delay transfusion preparation.

Freeze‐dried plasma (FDP) offers logistical advantages over FFP and has historically been used mainly in a military context, with limited access in civilian healthcare. FDP is commonly produced through lyophilization, although spray drying is an alternative technology. Depending on the manufacturer, FDP may be derived from pooled donor plasma or single‐donor units, and some products undergo pathogen reduction while others do not.^13^, ^14^, ^15^, ^16^, ^17^, ^18^ Before use, FDP is reconstituted in sterile water and can be prepared within approximately 10 min. Unlike FFP, FDP can be stored at ambient temperatures, making it highly suitable for austere or resource‐limited environments. In vitro studies have demonstrated that FDP maintains comparable quality to FFP, with only a moderate (~10%) reduction in labile clotting factors and no increased risk of adverse reactions.^14^, ^15^, ^16^, ^17^, ^18^, ^19^, ^20^, ^21^ Given the advantages, FDP represents an alternative resuspension medium for cryopreserved platelets, particularly in military or remote settings. A previous study reported promising results with French Lyophilized Plasma (FLyP),^22^ but the impact of other FDP formulations on platelet phenotype and function is not investigated. To address this, our study aims to compare the effects of OctaplasLG Powder and FFP as reconstitution media on the quality and function of cryopreserved platelets in vitro.

## 2 MATERIALS AND METHODS

### 2.1 Cryopreservation

Cryopreserved platelets were prepared from double‐dose platelet concentrates (*n* = 8), produced from pools of eight ABO‐identical buffy coats, the day after collection, as previously described.^23^ These double‐dose platelets are suspended in about 65% additive solution (SSP+) and 35% plasma. Each double‐dose concentrate was evenly divided into two separate freezing bags (Macopharma, Tourcoing, France) using a sterile welding device (Terumo BCT, Lakewood, CO, USA). Thereafter, freezing medium containing 25% DMSO in NaCl (9 mg/mL; 50 mL) was sterilely added to each platelet concentrate (*n* = 16). The platelet concentrates were then centrifuged at 1200 × g for 10 min, after which nearly all the supernatant was removed. Resulting in an approximately 10‐mL frozen platelet suspension in 5% DMSO. The prepared units were immediately put in metal boxes (Ninolab, Stockholm, Sweden) and placed in a −80°C freezer, subsequently stored for 29–210 days.

### 2.2 Plasma preparation

FFP (AB = 8) was prepared from whole blood donations at Karolinska University Hospital, processed, and frozen at ≤ − 25 °C within 16 h of collection. For reconstitution, FFP was thawed at 37°C for ~20 min using a Barkey plasma thawer (Barkey GmbH & Co. KG, Griesheim, Germany).

Lyophilized FDP, OctaplasLG Powder (AB = 8) (Octapharma, Lachen, Switzerland), is a mega‐pooled and solvent/detergent‐treated product.^14^ FDP was kept at +4°C until use and reconstituted in 200‐mL sterile water with a spike‐and‐needle set, as previously described.^13^

### 2.3 Thawing of platelets

Paired platelet units were thawed simultaneously using a Barkey plasma thawer (Barkey GmbH & Co. KG, Griesheim, Germany) at 37°C for <5 min. Each platelet unit was sterile welded (Terumo BCT, Lakewood, CO, USA) and resuspended in either 200 mL of FFP (Karolinska University Hospital) or 200 mL of FDP (Octaplas LG Powder), resulting in a final DMSO concentration of less than 1%. All units were gently massaged to dissolve aggregates and then maintained at room temperature without agitation for 30 min before further testing.

### 2.4 Intra‐ and extracellular metabolic parameters

Platelet counts and mean platelet volumes were measured using the hematology analyzer Swelab Alfa (Boule Medical, Stockholm, Sweden). Blood gas parameters, including pH, glucose, lactate, bicarbonate, CO_2_, and O_2_, along with electrolytes (cNa^+^, cK^+^, and cCl^−^), were analyzed using the ABL 800 (Radiometer Medical ApS, Copenhagen, Denmark). To assess platelet lysis, extracellular lactate dehydrogenase (LDH) activity was measured via spectrophotometry using an assay kit (Sigma‐Aldrich N6660, St. Louis, USA). Samples from the supernatant (centrifuged for 15 min at 3000 rpm) and platelet units (pretreated 1:10 with 0.5% Triton) were prepared and frozen at −80°C in phosphate buffer. Upon analysis, absorbance readings were recorded at 340 nm at 1‐minute intervals over 3 min using the UV5Bio spectrophotometer (Mettler Toledo, Greifensee, Switzerland). LDH activity was calculated and expressed as a percentage of total available activity.

### 2.5 Flow cytometry

The expression of various platelet markers was analyzed by flow cytometry. Samples were single stained with CD41‐FITC (Beckman Coulter Immunotech, Marseille, France); CD42b‐FITC (Beckman Coulter Immunotech, Marseille, France); CD61‐PE (Beckman Coulter Immunotech, Marseille, France); CD62P‐PE (Beckman Coulter Immunotech, Marseille, France); CD63‐FITC (Beckman Coulter Immunotech, Marseille, France); CD31‐FITC (Sigma‐Aldrich, St. Louis, MI, USA); GPVI‐PE (Pharmingen BD Biosciences, San Jose, CA, USA); PAC1‐FITC (BD Biosciences, California, USA); and Annexin V‐ FITC (BD Biosciences, Erembodegem, Belgium). The proportion (%) of positive cells was calculated from 5000 gated events, analyzed on the CytoFLEX Flow Cytometer (Beckman Coulter Life Sciences). Median fluorescence intensity (MFI) was calculated for the positive population of each marker, representing the MFI of positive events within the platelet gate.

Changes in the platelet mitochondria transmembrane potential (Δψ) were assessed using the mitochondrial permeability transition detection kit MitoPT JC‐1 (Immuno‐Chemistry Technologies, LCC, Bloomington, MN, USA), analyzed on CytoFLEX Flow Cytometer (Beckman Coulter Life Sciences). Dual‐color detection distinguished polarized (red fluorescence) from depolarized mitochondria (green fluorescence). The percentage of positive cells corresponds to the fraction with intact Δψ.

Platelet microparticles (PMPs) were analyzed directly from platelet units without centrifugation to minimize sample manipulation. Microparticles (<1 μm) were identified using size‐calibrated beads and dual staining with CD61‐PE (Beckman Coulter Immunotech, Marseille, France) and Annexin V‐FITC (BD Biosciences, Erembodegerm, Belgium), as previously described.^24^ For each sample, 10,000 gated events (<1 μm) were acquired. PMP proportion was based on CD61+ single‐positive or CD61+/Annexin V double‐positive events, and Annexin V+ fractions within CD61+ were calculated to assess phosphatidylserine exposure. Analyses were performed on a CytoFLEX Flow Cytometer (Beckman Coulter Life Sciences).

### 2.6 Soluble factors

Levels of VEGF‐A and sP‐selectin were quantified using the Ella™ microfluidic automated immunoassay system (Bio‐Techne, Minneapolis, USA), according to the manufacturer's instructions. In short, samples were stored at −80°C until batch analysis. Each batch also included a sample from a randomly selected FFP and FDP unit, which served as an indicative baseline control. Before analysis, samples were thawed and centrifuged at 10,000 × g for 5 min at 4°C. VEGF‐A levels were measured using 16‐sample cartridges, with samples diluted 1:2 in sample diluent. Levels of sP‐selectin were measured using 32‐sample cartridges, with samples diluted 1:100 in dilution buffer. For each assay, 50 μL of diluted sample was loaded into each well. Data acquisition was performed using the Simple Plex Runner Software v.4.1.0.22 (ProteinSimple).

### 2.7 Thromboelastometry

Thromboelastometry was performed using a ROTEM delta 3000 (TEM International, GmbH, Munich, Germany). A set volume of the platelet sample was mixed 1:1 with red cell concentrate and directly analyzed on the EXTEM and FIBTEM channels according to the manufacturer's instructions.

### 2.8 Statistics

The mean values and standard deviations are given if not further specified. Normality was assessed using the Shapiro–Wilk test. For normally distributed data, paired *t*‐tests were performed to determine statistical significance (*p* < .05). For non‐normally distributed data, differences were assessed using the Wilcoxon matched pairs signed rank test. For comparisons involving more than three parameters, multiple comparison correction was applied using the Holm‐Šidák method. All the statistical analyses were carried out using GraphPad Prism version 10.0.3.

## 3 RESULTS

### 3.1 Operational assessment of preparing the platelets

The thawing time for FFP was about 20 min, while the reconstitution of FDP was completed in less than 10 min. All FDP units dissolved easily, produced an acceptable amount of foam, and showed no visible aggregates. The preparation time for cryopreserved platelets was consistent regardless of whether FFP or FDP was used, with all units processed within 10 min, typically around 5 min. No significant differences in aggregate formation were observed. Only one paired unit per arm showed increased presence of small aggregates, which resolved with gentle manual agitation.

### 3.2 Improved platelet recovery in FDP‐reconstituted units

**FIGURE 1:**
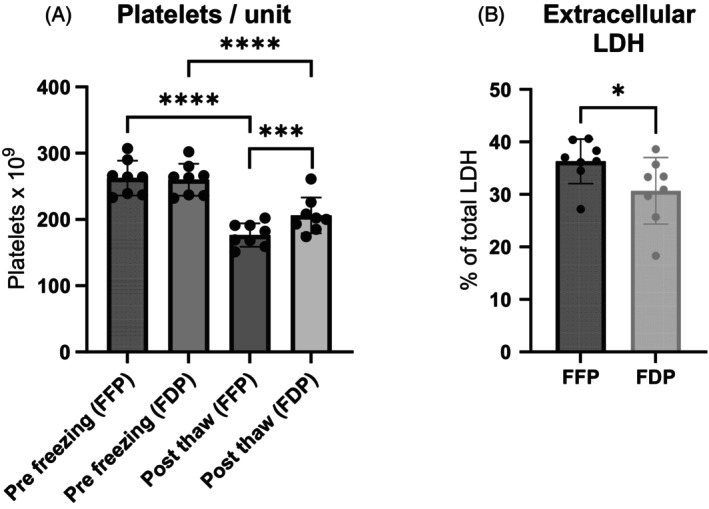
Improved platelet recovery in FDP units. Platelet integrity following reconstitution with FFP or FDP was assessed using platelet count (A) and extracellular LDH (B). Both assays demonstrated significant differences, with FDP showing a higher platelet concentration and lower LDH extracellularly compared to FFP. Data are presented as bars (mean ± SD) with dots representing each unit (*n* = 8). *p < .05, **p < .001, ***p < .0001.

The platelet count per unit was similar in both groups before freezing, with a mean of 262 ± 26 × 10^9^ (FFP) and 260 ± 24 × 10^9^ (FDP). After freezing, the units reconstituted with FDP contained significantly more platelets, 206 ± 27 × 10^9^/unit, compared to those reconstituted with FFP, 176 ± 18 × 10^9^/unit (*p* = .0006) (Figure 1A). Accordingly, the average platelet recovery was 79% for FDP and 67% for FPP. Extracellular LDH was lower in the FDP group, with a mean of 31% ± 6%, compared to 36% ± 4% in the FFP group (*p* = .040) (Figure 1B). The mean platelet volume (MPV) also differed significantly, with the FDP platelets being larger than those in the FFP group, 9.8 ± 0.3 vs. 9.5 ± 0.3 (*p* = .003).

### 3.3 Differences in oxygen and carbon dioxide pressure

In general, most blood gas parameters, including pH, remained comparable between FFP and FDP (Table 1). However, a notable difference was observed in oxygen and carbon dioxide pressure. The FFP platelets contained significantly more carbon dioxide (*p* < .0001) and less oxygen (*p* < .001) than FDP platelets. Additionally, reconstitution with FFP was associated with higher glucose and bicarbonate levels compared to FDP (*p* < .0001).

**TABLE 1:** Blood gas and electrolytes after reconstitution. Data presented as mean ± SD.

	FFP *n* = 8	FDP *n* = 8	*p*‐value (adjusted)
pH (37°C)	6.989 ± 0.029	7.003 ± 0.011	.544
pCO_2_ (kPa)	10.28 ± 0.49	1.72 ± 0.27	<.0001*
pO_2_ (kPa)	11.9 ± 2.0	17.5 ± 2.8	.005*
K+ (mmol/L)	4.0 ± 0.2	4.1 ± 0.1	.179
Na+ (mmol/L)	151 ± 4.8	146 ± 1.8	.097
Cl− (mmol/L)	86 ± 6.0	83 ± 0.9	.574
Glucose (mmol/L)	16.4 ± 2.2	4.2 ± 0.3	<.0001*
Lactate (mmol/L)	2.0 ± 1.1	1.9 ± 0.1	.814
Bicarbonate (mmol/L)	17.7 ± 1.2	3.1 ± 0.4	<.0001*

*Note*: Bold formatting is applied to all significant *p*‑values from *p < 0.05.

### 3.4 Similar phenotypic expression

**FIGURE 2:**
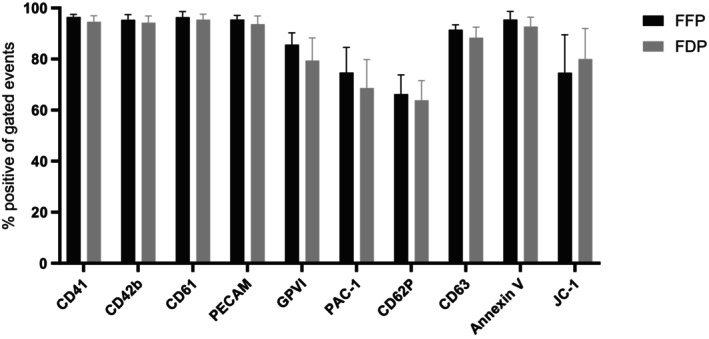
FDP use did not affect platelet phenotype compared to FFP. The phenotypic expression of extracellular and intracellular structures was assessed by flow cytometry in platelets reconstituted in either FFP or FDP. No significant differences were observed between the groups (*p* > .05). Data are presented as mean ± SD (error bars) (*n* = 8).

The expression of various platelet‐specific receptors involved in aggregation, adhesion, and activation did not differ significantly between FDP and FFP units when assessed by both the proportion of positive events (%) (Figure 2) and median fluorescence intensity (MFI) (Table 2). Platelets reconstituted in both FFP and FDP exhibited high levels of spontaneous activation, with over 60% positivity as determined by CD62P and PAC‐1. Likewise, phosphatidylserine expression was elevated in both groups, exceeding 90%. Staining of the mitochondrial membrane potential (ΔΨ) by JC‐1 showed no significant difference, with a mean of 74% ± 15% for FFP and 80% ±12% for FDP (Figure 2).

**TABLE 2:** Flow cytometric assessment of platelet phenotype in FDP and FFP units.

	FDP *n* = 8		FFP *n* = 8		*p*‐value (adjusted)	
	% positive gated events	Median MFI	% positive gated events	Median MFI	% positive gated events	Median MFI
CD41	96.5 ± 1.1	427 k ± 21 k	94.6 ± 2.3	429 k ± 41 k	.68	>.99
CD61	96.4 ± 2.2	548 k ± 70 k	94.3 ± 2.1	599 k ± 60 k	.58	.39
CD42b	95.4 ± 2.1	79 k ± 15 k	94.3 ± 2.7	71 k ± 16 k	.74	.068
PECAM	95.5 ± 1.6	34 k ± 4.5 k	93.7 ± 3.2	39 k ± 2.7 k	.68	.15
GPVI	85.6 ± 4.7	11 k ± 4.2 k	79.4 ± 8.9	10 k ± 3.5 k	.08	.44
CD63	91.5 ± 2.0	16 k ± 2.1 k	88.4 ± 4.0	15 k ± 2.4 k	.19	.79
CD62p	66.2 ± 7.5	9.3 k ± 1.5 k	63.9 ± 7.7	9.9 k ± 1.4 k	.68	.76
PAC‐1	74.7 ± 9.9	5.1 k ± 0.4 k	68.6 ± 11.1	4.5 k ± 0.9 k	.68	.47
Annexin V	95.4 ± 3.2	324 k ± 127 k	92.7 ± 3.7	250 k ± 91 k	.48	.12
JC‐1 (ΔΨm)	74.6 ± 14.9	n/a	80.0 ± 11.9	n/a	.68	n/a

*Note*: Median MFI calculated for positive gated events; values represent mean ± SD for % positive events.

### 3.5 Release of soluble factors and microparticles

**FIGURE 3:**
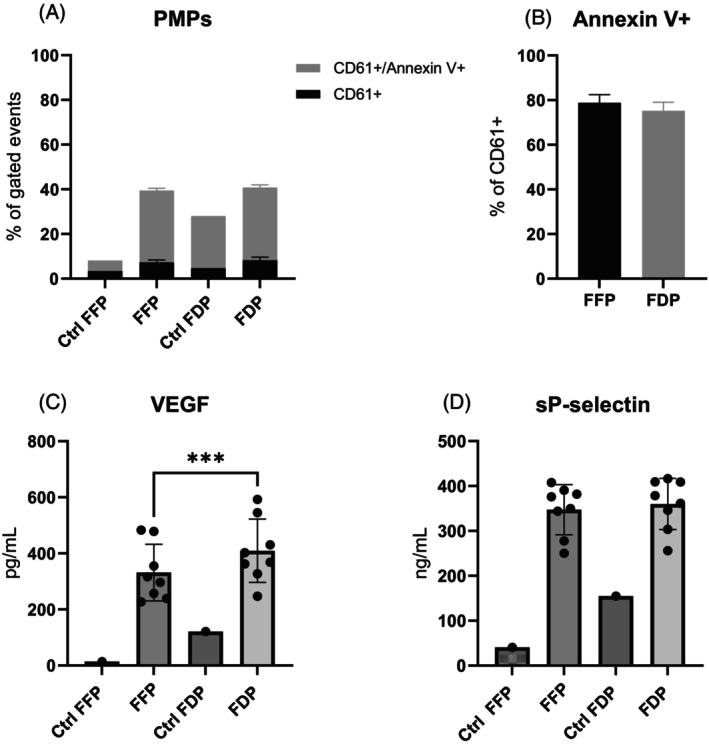
Microparticles and α‐granule secretion in the reconstituted cryopreserved platelets. (A) PMPs were analyzed by flow cytometry and categorized as CD61^+^/Annexin V^+^ (double‐positive) or CD61^+^ only (single‐positive). (B) The proportion of Annexin V^+^ events within CD61^+^ microparticles was compared between FFP and FDP groups. (C) VEGF and (D) soluble P‐selectin concentrations were measured in plasma supernatants. Each group included one control plasma unit (Ctrl FFP and Ctrl FDP) as indicative examples of baseline values. Data are presented as mean ± SD (error bars) (*n* = 8 platelet units per group; control plasma shown as single bars). ***p < .0001.

Flow cytometric analysis of platelet‐derived microparticles revealed no significant differences between FFP and FDP reconstitution of cryopreserved platelets. The proportion of CD61+ microparticles was similar between groups, averaging 32% in the FFP group and 33% in the FDP group (Figure 3A). The proportion of phosphatidylserine‐positive (annexin V+) particles among CD61+ microparticles was also consistent, with a mean of 79% in the FFP group and 75% in the FDP group (Figure 3B). Additionally, one control plasma sample was analyzed, indicating a higher presence of CD61^+^ PMPs in FDP (23%) compared to FFP (5%). Phosphatidylserine expression also appeared higher in FDP control plasma (89%) than in FFP (56%).

In addition, the release of VEGF and sP‐selectin was measured in the two groups, along with one randomly selected control unit per group, to indicate baseline values. The concentration of VEGF was significantly higher in the FDP group compared to the FFP, at 409 ± 113 pg/mL versus 331 ± 101 pg/mL, respectively (*p* = .0003) (Figure 3C). In contrast, sP‐selectin concentrations were statistically comparable, with mean values of 360 ± 57 ng/mL for FDP and 347 ± 56 ng/mL for FFP (Figure 3D). Notably, FDP control plasma contained higher concentrations of these factors than FFP control plasma, suggesting that a smaller proportion of the measured concentrations in the FDP group originated from platelets rather than from the plasma itself.

### 3.6 Faster clotting time (CT) in FDP units

**FIGURE 4:**
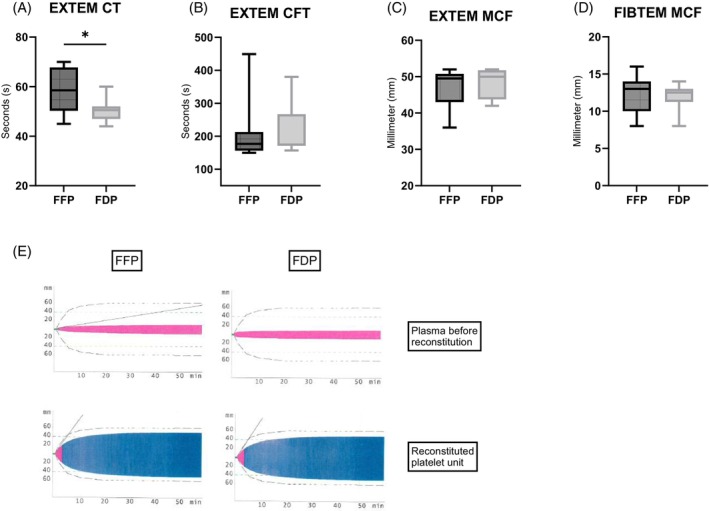
Reduced clotting time in FDP units. Thromboelastography using the EXTEM channel assessed (A) clotting time, (B) clot formation time, and (C) maximum clot firmness for cryopreserved platelets reconstituted with either FFP or FDP. (D) Maximum clot firmness using the FIBTEM channel. (E) Representative ROTEM EXTEM graphs for FFP and FDP groups: upper panels show plasma alone before reconstitution; lower panels show reconstituted platelet units. Data are presented as box‐and‐whisker plots (5th–95th percentile) (*n* = 8). *p < .05.

ROTEM was used to assess the in vitro coagulation capacity of the thawed platelets. The use of FDP significantly shortened the EXTEM clotting time (CT) compared to FFP. The mean CT for FDP was 50 ± 5 s, while for FFP it was 58 ± 9 s (*p* = .035) (ref. 38–79 s) (Figure 4A). However, no significant differences were observed in EXTEM clot formation time (CFT) or maximum clot firmness (MCF) (Figure 4B,C). The average CFT exceeded the reference range < 159 s, with mean values around 210 s in both groups. Similarly, MCF was slightly below the reference threshold of >50 mm, with a mean value of 48 mm in both groups. In addition, MCF values assessed using the FIBTEM channel showed no significant differences between groups, 12 ± 3 mm for FFP and 12 ± 2 mm for FDP (ref. 9–25 mm) (Figure 4D). The platelet‐dependent contribution to clot strength, calculated as the difference between EXTEM MCF and FIBTEM MCF, did not differ significantly between groups (FFP: 34.8 ± 3.5; FDP: 36.5 ± 5.0; *p* = .1602).

## 4 DISCUSSION

Utilizing FDP as a reconstitution medium may facilitate the availability of cryopreserved platelets and potentially offer an advantage in emergency transfusion scenarios. In this study, we compared the functional characteristics of cryopreserved platelets reconstituted in FDP (Octaplas LG Powder) and in‐house FFP. Interestingly, reconstitution with FDP resulted in higher platelet recovery and lower extracellular LDH activity, indicating improved structural integrity. These findings are consistent with previous reports suggesting FDP as a feasible alternative, though not necessarily superior to FFP.^22^

The observed improvement in platelet recovery is noteworthy, though the underlying mechanism remains unclear. After reconstitution, FDP units exhibited lower CO_2_ levels compared to FFP units. While elevated CO_2_ is associated with pH reduction and impaired platelet viability during storage,^25^, ^26^ this typically affects long‐term storage rather than short‐term reconstitution. Both groups had pH values above 6.4, as recommended by the Council of Europe,^27^ making CO_2_‐driven pH changes unlikely to account for the difference. All analyses were performed shortly after reconstitution, so potential effects of prolonged storage remain unassessed. Furthermore, mitochondrial membrane potential was comparable between groups, indicating that no major metabolic disruption occurred under the studied conditions. Instead, the observed reduction in platelet counts points toward non‐mitochondrial mechanisms.

Bearing that in mind, it is plausible that only the short‐term exposure during reconstitution could transiently impact platelet integrity. Hypothetically, the higher recovery observed in the FDP units may be attributed to the composition of FDP, creating a more protective or stabilizing environment for platelets following reconstitution. Differences in protein content, osmolality, or ionic strength could influence platelet stability, aggregation tendency, or susceptibility to non‐apoptotic lysis.^28^ The FDP environment may therefore help reduce platelet loss by limiting aggregation or lysis, potentially offering a more favorable condition post‐reconstitution.

While FDP appears to support platelet integrity, its impact on platelet phenotype was further investigated. It is widely recognized that cryopreservation significantly alters platelet morphology, leading to the emergence of distinct subpopulations, including activated, procoagulant, and novel phenotypes.^29^, ^30^ These alterations are partly characterized by increased expression of activation markers such as P‐selectin, along with slight reductions in key surface receptors like CD42b (GPIbα) and GPVI, while the overall expression of CD61 (GPIIb/IIIa) remains largely unchanged.^24^, ^31^, ^32^ Our findings align with previous reports, although the observed effects appear comparatively mild. The phenotypic patterns were highly consistent across both plasma types, reinforcing the feasibility of using FDP as a viable alternative for platelet reconstitution.

Cryopreservation not only alters platelet morphology but also triggers granule secretion, a process that shapes platelet function and their interactions with other cells. Among these, sP‐selectin and VEGF are modulators released from α‐granules associated with platelet activation, a process known to be elevated in cryopreserved platelets.^29^, ^33^, ^34^, ^35^ When comparing the two groups, VEGF levels were significantly higher in the FDP units, presumably due to the naturally higher VEGF content in FDP. Similarly, sP‐selectin levels were also initially higher in FDP; however, no difference was observed after platelet reconstitution. Suggesting that the release of sP‐selectin post‐reconstitution may be lower when using FDP. Consequently, this indicates that FDP may contribute to a lower release of intracellular substances, supporting earlier conclusions that reconstitution in FDP reduces cell disintegration in cryopreserved platelets.

A major limitation of this study is that only one randomly selected plasma from each study group was measured as a control. This provides illustrative rather than representative data and does not account for donor‐to‐donor variability in FFP. Ideally, all plasma units should have been tested prior to reconstitution to accurately assess baseline levels of biological modulators and their potential impact on platelet integrity. An increase in biological modulators could potentially contribute to adverse immunomodulatory effects in the recipient,^35^, ^36^, ^37^ underscoring the need for systematic pre‐reconstitution testing in future studies. Furthermore, this limitation extends to include other study parameters such as LDH, microparticles, blood gases, and ROTEM. Without testing plasma prior to reconstitution, it is not possible to provide a comprehensive understanding of plasma composition and its influence on platelet recovery, phenotype, and function.

To assess whether platelet clotting function remained effective after reconstitution, ROTEM analysis was performed. Despite the higher platelet count in FDP units, no improvement in clot strength was observed in the EXTEM channel. Both EXTEM CFT and MCF were similar between the groups, indicating comparable overall clotting capacity. However, CFT and MCF values in both groups were outside the normal reference range, consistent with previous studies showing reduced clot strength compared to liquid‐stored platelets.^31^, ^38^, ^39^, ^40^ Prolonged CFT and reduced MCF in EXTEM may also be attributed to decreased levels of fibrinogen or factor XIII.^41^, ^42^ Notably, FIBTEM MCF, which assesses clot firmness independent of platelet contribution, remained within the normal range. This suggests that EXTEM impairment is more likely due to platelet dysfunction rather than deficiencies in plasma coagulation factors. This is further supported by earlier studies, demonstrating similar levels of FXIII and fibrinogen in FDP and FFP.^13^, ^14^

Interestingly, FDP was associated with a significantly shorter EXTEM CT, indicating accelerated clot initiation. This effect may be attributed to differences in the composition of plasma coagulation factors, which can influence the initiation phase of clot formation.^43^ Octaplas LG Powder, a pooled plasma product, offers more consistent quality, whereas FFP, derived from single donors, exhibits greater variability, as reflected in our results. However, in a previous study,^13^ we found no difference in clot initiation between Octaplas LG Powder and FFP. Notably, that study employed TEG rather than ROTEM, which may account for the discrepancy. Similarly, another study^44^ also failed to demonstrate a shortened CT when comparing Octaplas LG Powder to FFP. Yet, the use of only three FFP units in that study may have limited its ability to detect donor variability.

An alternative explanation for the shortened CT could be the presence of procoagulant platelet‐derived particles, which are believed to enhance coagulation initiation.^45^, ^46^, ^47^ However, in the present study, we found no significant differences in the proportion of microparticles between the groups, nor did we observe any differences in phosphatidylserine expression, both of which are considered contributors to procoagulant activity. However, the single control plasma analyzed from the FDP group appeared to contain more PMPs than its FFP counterpart. Although this observation is not representative of the entire group, it suggests that plasma composition may influence PMP levels and warrants further investigation, including assessment of the total number of PMPs rather than just their proportion.

So far, only a limited number of studies have explored reconstitution media other than plasma. Two studies have examined the use of platelet additive solutions, SSP+ and PAS‐G, with supportive results.^12^, ^48^ In the study by Johnson et al.,^12^ platelets resuspended in glucose‐containing media, such as PAS‐G or plasma, demonstrated better recovery and in vitro quality than those resuspended in glucose‐free SSP+. This supports previous assumptions that glucose is essential for optimal platelet storage.^49^ Another study reconstituted cryopreserved platelets in saline, which resulted in lower in vitro recovery but acceptable in vivo survival after transfusion.^10^ The lack of studies highlights the need for further research to identify the most effective resuspension methods. To date, FFP has remained the preferred medium, primarily due to its hemostatic properties, which are critical in managing bleeding trauma.^50^, ^51^, ^52^

In summary, this study demonstrates that FDP is a viable alternative to FFP for the reconstitution of cryopreserved platelets. FDP was associated with faster clot initiation and improved platelet recovery, as indicated by higher platelet counts and lower extracellular LDH activity. While the underlying mechanisms remain to be fully elucidated, differences in plasma composition, such as blood gas parameters or coagulation factor consistency, may contribute to the observed effects. Importantly, FDP offers significant logistical advantages, particularly in remote resource‐limited settings, by reducing preparation time and eliminating the need for thawed plasma. The study findings support the potential implementation of FDP as a reconstitution medium, particularly in settings where rapid availability and logistical efficiency are critical.

## FUNDING INFORMATION

Research grant (ALF) from the Stockholm County, no 530783.

## CONFLICT OF INTEREST STATEMENT

The authors have disclosed no conflicts of interest.

## Data Availability

The data that support the findings of this study are available from the corresponding author upon reasonable request.

## References

[1] Kelly K , Cancelas JA , Szczepiorkowski ZM , Dumont DF , Rugg N , Dumont LJ . Frozen platelets‐development and future directions. Transfus Med Rev. 2020;34(4):286–293. 10.1016/j.tmrv.2020.09.008 33317698

[2] Noorman F , Rijnhout TWH , de Kort B , Hoencamp R . Frozen for combat: quality of deep‐frozen thrombocytes, produced and used by The Netherlands armed forces 2001‐2021. Transfusion. 2023;63(1):203–216. 10.1111/trf.17166 36318083 PMC10092739

[3] Holley A , Marks DC , Johnson L , Reade MC , Badloe JF , Noorman F . Frozen blood products: clinically effective and potentially ideal for remote Australia. Anaesth Intensive Care. 2013;41(1):10–19. 10.1177/0310057X1304100104 23362885

[4] Noorman F , van Dongen TT , Plat MJ , Badloe JF , Hess JR , Hoencamp R . Transfusion: −80°C frozen blood products are safe and effective in military casualty care. PLoS One. 2016;11(12):e0168401. 10.1371/journal.pone.0168401 27959967 PMC5154589

[5] Wikman A , Diedrich B , Björling K , Forsberg P‐O , Harstad A‐M , Henningsson R , et al. Cryopreserved platelets in bleeding management in remote hospitals: a clinical feasibility study in Sweden. Front Public Health. 2023;10:1073318. 10.3389/fpubh.2022.1073318 36743180 PMC9894868

[6] Kleinveld DJB , Sloos PH , Noorman F , Maas MAW , Kers J , Rijnhout TWH , et al. The use of cryopreserved platelets in a trauma‐induced hemorrhage model. Transfusion. 2020;60(9):2079–2089. 10.1111/trf.15937 32592423 PMC7540664

[7] Reade MC , Marks DC , Bellomo R , Deans R , Faulke DJ , Fraser JF , et al. A randomized, controlled pilot clinical trial of cryopreserved platelets for perioperative surgical bleeding: the CLIP‐I trial (editorial, p. 2759). Transfusion. 2019;59(9):2794–2804. 10.1111/trf.15423 31290573

[8] Valeri CR , Ragno G , Khuri S . Freezing human platelets with 6 percent dimethyl sulfoxide with removal of the supernatant solution before freezing and storage at −80 degrees C without postthaw processing [published correction appears in transfusion. 2006 Feb;46(2):313]. Transfusion. 2005;45(12):1890–1898. 10.1111/j.1537-2995.2005.00647.x 16371041

[9] Waters L , Marks DC , Johnson L . Strategies to improve platelet cryopreservation: a narrative review. Transfusion. 2025;65(4):740–749. 10.1111/trf.18204 40059666 PMC12005584

[10] Dumont LJ , Cancelas JA , Dumont DF , Siegel AH , Szczepiorkowski ZM , Rugg N , et al. A randomized controlled trial evaluating recovery and survival of 6% dimethyl sulfoxide‐frozen autologous platelets in healthy volunteers. Transfusion. 2013;53(1):128–137. 10.1111/j.1537-2995.2012.03735.x 22671278

[11] Hess JR , Holcomb JB . Transfusion practice in military trauma. Transfus Med. 2008;18(3):143–150. 10.1111/j.1365-3148.2008.00855.x 18598276

[12] Johnson L , Reid S , Tan S , Vidovic D , Marks DC . PAS‐G supports platelet reconstitution after cryopreservation in the absence of plasma. Transfusion. 2013;53(10):2268–2277. 10.1111/trf.12084 23347144

[13] Ehn K , Skallsjö G , Romlin B , Sandström G , Sandgren P , Wikman A . An experimental comparison and user evaluation of three different dried plasma products. Vox Sang. 2025;120:1058–1065. 10.1111/vox.13798 39870389 PMC12602137

[14] Heger A , Gruber G . Frozen and freeze‐dried solvent/detergent treated plasma: two different pharmaceutical formulations with comparable quality. Transfusion. 2022;62(12):2621–2630. 10.1111/trf.17139 36181447 PMC10092463

[15] Liu QP , Carney R , Sohn J , Sundaram S , Fell MA . Single‐donor spray‐dried plasma. Transfusion. 2019;59(2):707–713. 10.1111/trf.15035 30443975

[16] Martinaud C , Civadier C , Ausset S , Verret C , Deshayes AV , Sailliol A . In vitro hemostatic properties of French lyophilized plasma. Anesthesiology. 2012;117(2):339–346. 10.1097/ALN.0b013e3182608cdd 22739764

[17] Bux J , Dickhörner D , Scheel E . Quality of freeze‐dried (lyophilized) quarantined single‐donor plasma. Transfusion. 2013;53(12):3203–3209. 10.1111/trf.12191 23581390

[18] Cancelas JA , Nestheide S , Rugg N , Eckerman A , Macdonald VW , L Charles M , et al. Characterization and first‐in‐human clinical dose‐escalation safety evaluation of a next‐gen human freeze‐dried plasma. Transfusion. 2022;62(2):406–417. 10.1111/trf.16756 34951486 PMC9306459

[19] Sailliol A , Martinaud C , Cap AP , Civadier C , Clavier B , Deshayes A‐V , et al. The evolving role of lyophilized plasma in remote damage control resuscitation in the French armed forces health service. Transfusion. 2013;53(1):65S–71S. 10.1111/trf.12038 23301975

[20] Sunde GA , Vikenes B , Strandenes G , Flo KC , Hervig TA , Kristoffersen EK , et al. Freeze dried plasma and fresh red blood cells for civilian prehospital hemorrhagic shock resuscitation. J Trauma Acute Care Surg. 2015;78(6 Suppl 1):S26–S30. 10.1097/TA.0000000000000633 26002260

[21] Gokhale SG , Scorer T , Doughty H . Freedom from frozen: the first British military use of lyophilised plasma in forward resuscitation. J R Army Med Corps. 2016;162(1):63–65. 10.1136/jramc-2014-000361 25535320

[22] Martinaud C , Sugier HHR , Javaudin O , Burin des Roziers N , Bégué S . In vitro characteristics of cryopreserved platelet concentrates reconstituted by fresh frozen or lyophilized plasma. Transfus Clin Biol. 2022;29(2):118–123. 10.1016/j.tracli.2022.01.003 35032661

[23] Ohlsson S , Diedrich B , Uhlin M , Sandgren P . Optimized processing for pathogen inactivation of double‐dose Buffy‐coat platelet concentrates: maintained in vitro quality over 7‐day storage. Vox Sang. 2018;113:611–621. 10.1111/vox.12696 30156292

[24] Ehn K , Wikman A , Uhlin M , Sandgren P . Cryopreserved platelets in a non‐toxic DMSO‐free solution maintain hemostatic function in vitro. Int J Mol Sci. 2023;24(17):13097. 10.3390/ijms241713097 37685902 PMC10488190

[25] Murphy S , Gardner FH . Platelet storage at 22 degrees C: role of gas transport across plastic containers in maintenance of viability. Blood. 1975;46(2):209–218. 237590

[26] Skripchenko A , Myrup A , Thompson‐Montgomery D , Awatefe H , Wagner SJ . Mitochondrial dysfunction of platelets stored in first‐ and second‐generation containers is, in part, associated with elevated carbon dioxide levels. Transfusion. 2011;51(2):371–379. 10.1111/j.1537-2995.2010.02829.x 20796252

[27] European Directorate for the Quality of Medicines & HealthCare . Guide to the preparation, use and quality Assurance of Blood Components. 22nd ed. Strasbourg: Council of Europe; 2025.

[28] Pieters M , Jerling JC , Weisel JW . Effect of freeze‐drying, freezing and frozen storage of blood plasma on fibrin network characteristics. Thromb Res. 2002;107(5):263–269. 10.1016/s0049-3848(02)00344-4 12479888

[29] Johnson L , Reade MC , Hyland RA , Tan S , Marks DC . In vitro comparison of cryopreserved and liquid platelets: potential clinical implications. Transfusion. 2015;55(4):838–847. 10.1111/trf.12915 25371169

[30] Lozano M , Cid J . Cryopreserved platelets: a narrative review of its current role in transfusion therapy. Ann Blood. 2021;7:40. 10.21037/aob-21-31

[31] Tynngård N , Bell A , Gryfelt G , Cvetkovic S , Wikman A , Uhlin M , et al. Cryopreservation of buffy coat derived platelets: paired in vitro characterization using uncontrolled versus controlled freezing rate protocols. Transfusion. 2021;61(2):546–556. 10.1111/trf.16227 33345368 PMC7898315

[32] Johnson L , Vekariya S , Tan S , Padula MP , Marks DC . Extended storage of thawed platelets: refrigeration supports postthaw quality for 10 days. Transfusion. 2020;60(12):2969–2981. 10.1111/trf.16127 33085783

[33] Michelson AD , Barnard MR , Hechtman HB , MacGregor H , Connolly RJ , Loscalzo J , et al. In vivo tracking of platelets: circulating degranulated platelets rapidly lose surface P‐selectin but continue to circulate and function. Proc Natl Acad Sci USA. 1996;93(21):11877–11882. 10.1073/pnas.93.21.11877 8876231 PMC38152

[34] Ferroni P , Martini F , Riondino S , La Farina F , Magnapera A , Ciatti F , et al. Soluble P‐selectin as a marker of in vivo platelet activation. Clin Chim Acta. 2009;399(1–2):88–91. 10.1016/j.cca.2008.09.018 18835553

[35] Edvardsen L , Taaning E , Dreier B , Christensen LD , Mynster T , Nielsen HJ . Extracellular accumulation of bioactive substances during preparation and storage of various platelet concentrates. Am J Hematol. 2001;67(3):157–162. 10.1002/ajh.1099 11391711

[36] Cognasse F , Hamzeh‐Cognasse H . Platelet‐derived immune‐modulatory mediators and transfusion: time to consider their effects? Blood Transfus. 2022;20(3):177–179. 10.2450/2022.0322-21 35302482 PMC9068357

[37] Morrell CN . Immunomodulatory mediators in platelet transfusion reactions. Hematology Am Soc Hematol Educ Program. 2011;2011:470–474. 10.1182/asheducation-2011.1.470 22160076

[38] Waters L , Padula MP , Marks DC , Johnson L . Calcium chelation: a novel approach to reduce cryopreservation‐induced damage to frozen platelets. Transfusion. 2020;60(7):1552–1563. 10.1111/trf.15799 32319689

[39] Six KR , Delabie W , Devreese KMJ , Johnson L , Marks DC , Dumont LJ , et al. Comparison between manufacturing sites shows differential adhesion, activation, and GPIbα expression of cryopreserved platelets. Transfusion. 2018;58(11):2645–2656. 10.1111/trf.14828 30312492

[40] Pérez‐Ferrer A , Navarro‐Suay R , Viejo‐Llorente A , Alcaide‐Martín MJ , de Vicente‐Sánchez J , Butta N , et al. In vitro thromboelastometric evaluation of the efficacy of frozen platelet transfusion. Thromb Res. 2015;136(2):348–353. 10.1016/j.thromres.2015.05.031 26058942

[41] Lier H , Vorweg M , Hanke A , Görlinger K . Thromboelastometry guided therapy of severe bleeding. Essener Runde algorithm. Hamostaseologie. 2013;33(1):51–61. 10.5482/HAMO-12-05-0011 23258612

[42] Dirkmann D , Görlinger K , Dusse F , Kottenberg E , Peters J . Early thromboelastometric variables reliably predict maximum clot firmness in patients undergoing cardiac surgery: a step towards earlier decision making. Acta Anaesthesiol Scand. 2013;57(5):594–603. 10.1111/aas.12040 23240733

[43] Theusinger OM , Schröder CM , Eismon J , Emmert MY , Seifert B , Spahn DR , et al. The influence of laboratory coagulation tests and clotting factor levels on rotation Thromboelastometry (ROTEM(R)) during major surgery with hemorrhage. Anesth Analg. 2013;117(2):314–321. 10.1213/ANE.0b013e31829569ac 23780419

[44] Shoara AA , Singh K , Peng HT , Moes K , Yoo J‐A , Sohrabipour S , et al. Freeze‐dried plasma: hemostasis and biophysical analyses for damage control resuscitation. Transfusion. 2025;65(1):S250–S264. 10.1111/trf.18124 39806922 PMC12035980

[45] Johnson L , Coorey CP , Marks DC . The hemostatic activity of cryopreserved platelets is mediated by phosphatidylserine‐expressing platelets and platelet microparticles. Transfusion. 2014;54(8):1917–1926. 10.1111/trf.12578 24527873

[46] Raynel S , Padula MP , Marks DC , Johnson L . Cryopreservation alters the membrane and cytoskeletal protein profile of platelet microparticles. Transfusion. 2015;55(10):2422–2432. 10.1111/trf.13165 26046916

[47] Sinauridze EI , Kireev DA , Popenko NY , Pichugin AV , Panteleev MA , Krymskaya OV , et al. Platelet microparticle membranes have 50‐ to 100‐fold higher specific procoagulant activity than activated platelets. Thromb Haemost. 2007;97(3):425–434. 17334510

[48] McLean C , McMillan L , Petrik J , Fraser AR , Morrison A . Cryopreserved platelets and their suitability in being re‐suspended in additive solution. Vox Sang. 2020;115(8):676–685. 10.1111/vox.12993 32966615

[49] Gulliksson H . Defining the optimal storage conditions for the long‐term storage of platelets. Transfus Med Rev. 2003;17(3):209–215. 10.1016/s0887-7963(03)00020-8 12881781

[50] Lammers DT , Holcomb JB . Damage control resuscitation in adult trauma patients: what you need to know. J Trauma Acute Care Surg. 2023;95(4):464–471. 10.1097/TA.0000000000004103 37735778

[51] Cannon JW . Hemorrhagic shock. N Engl J Med. 2018;378(4):370–379. 10.1056/NEJMra1705649 29365303

[52] Pusateri AE , Moore EE , Moore HB , le TD , Guyette FX , Chapman MP , et al. Association of Prehospital Plasma Transfusion with Survival in trauma patients with hemorrhagic shock when transport times are longer than 20 minutes: a post hoc analysis of the PAMPer and COMBAT clinical trials. JAMA Surg. 2020;155(2):e195085. 10.1001/jamasurg.2019.5085 31851290 PMC6990948

